# A synthetic male-specific sterilization system using the mammalian pro-apoptotic factor in a malaria vector mosquito

**DOI:** 10.1038/s41598-019-44480-0

**Published:** 2019-06-03

**Authors:** Daisuke S. Yamamoto, Megumi Sumitani, Katsumi Kasashima, Hideki Sezutsu, Hiroyuki Matsuoka, Hirotomo Kato

**Affiliations:** 1 Division of Medical Zoology, Department of Infection and Immunity, Jichi Medical University, Yakushiji, Shimotsuke, Tochigi Japan; 2 Transgenic Silkworm Research Unit, Division of Biotechnology, Institute of Agrobiological Sciences, National Agriculture and Food Research Organization (NARO), Owashi, Tsukuba, Ibaraki Japan; 3 Division of Functional Biochemistry, Department of Biochemistry, Jichi Medical University, Yakushiji, Shimotsuke, Tochigi Japan

**Keywords:** Transgenic organisms, Genetic engineering, Apoptosis, Spermatogenesis, Entomology

## Abstract

Conditional cell death systems are useful for various aspects of basic science with a wide range of applications, including genetic pest control. We recently demonstrated that expression of the mammalian pro-apoptotic factor, B-cell leukaemia/lymphoma 2-associated X protein (Bax), can induce apoptosis in specific tissues by using tissue specific promoters in silkworm and mosquito. Here, we newly identified a functional promoter in the Asian malaria vector, *Anopheles stephensi*, which enables gene expression specifically in the testis. We produced a transgenic mosquito line that expresses mouse Bax under the control of this testis-specific promoter. Transgenic mosquito males exhibited aberrant testes without functional sperm and complete sterility, whereas transgenic females maintained normal fecundity. Despite their abnormal testes, the transgenic males maintained normal function of male accessory glands and typical mating behaviour. As a result of mating with these males, females showed refractoriness to further mating. These results suggest that transgenic males induce female sterility via mating. The mosquito is one of the most important disease vectors, and the control of their population benefits global public health. Thus, this Bax-mediated synthetic male-specific sterilization system could be applied to population control of mosquitoes.

## Introduction

Insect pests are detrimental to health, agricultural production, and economic progress worldwide. Anopheline mosquitoes are competent vectors of malaria, an infectious disease that kills estimated 445,000 people each year worldwide and is an impactful global public health burden^1^. Mosquito vector control is one of the most effective methods to reduce malaria transmission. However, mosquitoes that are resistant to insecticides have recently been reported and impacted the effectiveness of insecticide sprays and insecticide-treated bed nets^2–4^. The genetic control of mosquitoes, such as population replacement or suppression with genetically engineered mosquitoes, has the potential to become a novel strategy for vector control. Recently, synthetic gene drive systems designed to spread a desired genetic trait into a population with super-Mendelian inheritance have been developed, and they have the potential to be effective vector control strategies^5–8^. In the anopheline mosquito, a homing CRISPR/Cas9-based gene drive for population replacement to a malaria resistant phenotype has been studied^9^. Gene drives also could be used to the method for the population suppression^10,11^. However, in homing CRISPR/Cas9-based gene-drives, it has been speculated that a non-homologous end joining error in the target allele DNA repair creates a drive-resistant allele and reduces spreading efficiency of the desired phenotype into populations^6^. Therefore, homing CRISPR/Cas9-based gene drives still need improvement^12,13^.

Another genetic population control strategy is the release of sterilized mosquito males to reduce the size of field populations, which known as the sterile insect technique (SIT). In classical SIT, male sterilization was attempted by irradiation or chemicals in many mosquito species^14^. However, irradiation has often caused reduced male mating competitiveness, thereby reducing the effect of SIT. Chemical sterilization has been controversial due to environmental contamination through the predator effect^15^. The genetic engineered systems for sterilization or carrying a dominant lethal trait in males is a promising alternative to irradiation or chemicals^14,16^. The gonad is an attractive target tissue to achieve the aim. Therefore, germline-specific promoters have promise for driving the expression of effector genes that cause sterility in germline cells. Previous studies report that the *beta2-tubulin* gene (*b2t*) promoter drives testis-specific expression of a transgene in mosquitoes and other dipteran species^17–20^. The *b2t* promoter of *An*. *gambiae* was also used for inducing dominant lethality on a gene-drive strategy. It is known that a homing endonuclease I-*Ppo*I specifically recognizes and cleaves the ribosomal DNA (rDNA) repeat sequences found solely on the X chromosome in *An*. *gambiae*^21^. In a previous study, I-*Ppo*I was combined with *b2t* promoter and expressed in the testes. By the expression of I-*Ppo*I in the testes, the shredding of paternal X-chromosome was caused and eggs were predominantly fertilized by Y-bearing sperm, thereby extreme male-biased progeny were produced^22^. Similarly, it was reported that expression system using Cas9 under the control of *b2t* promoter and a guide RNA that targeted the X-linked rDNA repeat sequences during spermatogenesis was applicable for induction of X-chromosome shredding and production of the male-biased progeny^23^. This X-chromosome shredding-based gene-drive system will be effective reducing the population. However, the X-linked rDNA repeat sequence was only conserved among *An*. *gambiae* complex species. The effect of X-chromosome shredding using I-*Ppo*I and CRISPR/Cas9 targeting X-linked rDNA would be limited to these species. Therefore, other target genes for X-chromosome shredding found in other mosquito species as well as pest other than mosquitoes are required.

In the present study, we aim to develop a new effector to induce sterility, and applied the mouse *B-cell leukaemia/lymphoma 2-associated X protein* gene (*mBax*) as an effector gene inducing sterility of males to testis-specific-expression using the *b2t* promoter in *An*. *stephensi*. Bax is a member of the Bcl-2 family in mammals, and mediates the mitochondrial outer membrane permeabilization, which is an essential event in cell death^24–26^. In the previous study, we demonstrated that mBax-mediated cell death induction is functional and effective in inhibiting the function of salivary glands in *An*. *stephensi*^27^. Here, we produced transgenic *An*. *stephensi* overexpressing mBax under the control of the *b2t* promoter. The males of the transgenic line had aberrant testes, and sperm were not observed in this tissue. We investigated the fertility of this transgenic mosquito and demonstrated that males are completely sterile. We also demonstrated that these males conferred mating refractoriness on females via mating. Therefore, mBax-mediated cell death during spermatogenesis can completely sterilize *An*. *stephensi* males and subsequently sterilize females. The mBax-mediated cell death induction system is functional in several different insect species^28,29^. Therefore, mBax could be a useful tool for mosquito control as well as overall insect management.

## Results

### Identification of the *beta2-tubulin* promoter in *An*. *stephensi*

*An*. *stephensi beta2-tubulin* gene (*Asb2t*) was identified in the genomic scaffold sequence of the VectorBase (https://www.vectorbase.org/) by using Protein BLAST. We used the *b2t* gene of *An*. *gambiae* (VectorBase accession number, AGAP008622) and *Ae*. *aegypti* (GenBank accession number, DQ833526) as the query sequences. In addition, we used the sequence feature found in the carboxyl terminus of insect testis specific-tubulin to identify *b2t*^30,31^. *Asb2t* (ASTE003208) is located on the genomic scaffold KB664744 and consists of a single exon encoding 446 amino acids (Supplementary Fig. S1A). The amino acid sequence of *Asb2t* was found to be 97.8% identical to the *An*. *gambiae* homologue. In order to confirm the testis specific-expression of *Asb2t*, we performed RT-PCR analysis in *An*. *stephensi*. Expression of *Asb2t* was confirmed specifically in male pupae and testes of adult males (Supplementary Fig. S1B). The expression was not detected in female pupae and adults. This expression profile is similar to that of other dipteran species, as previously reported^18,32^.

**Figure 1:**
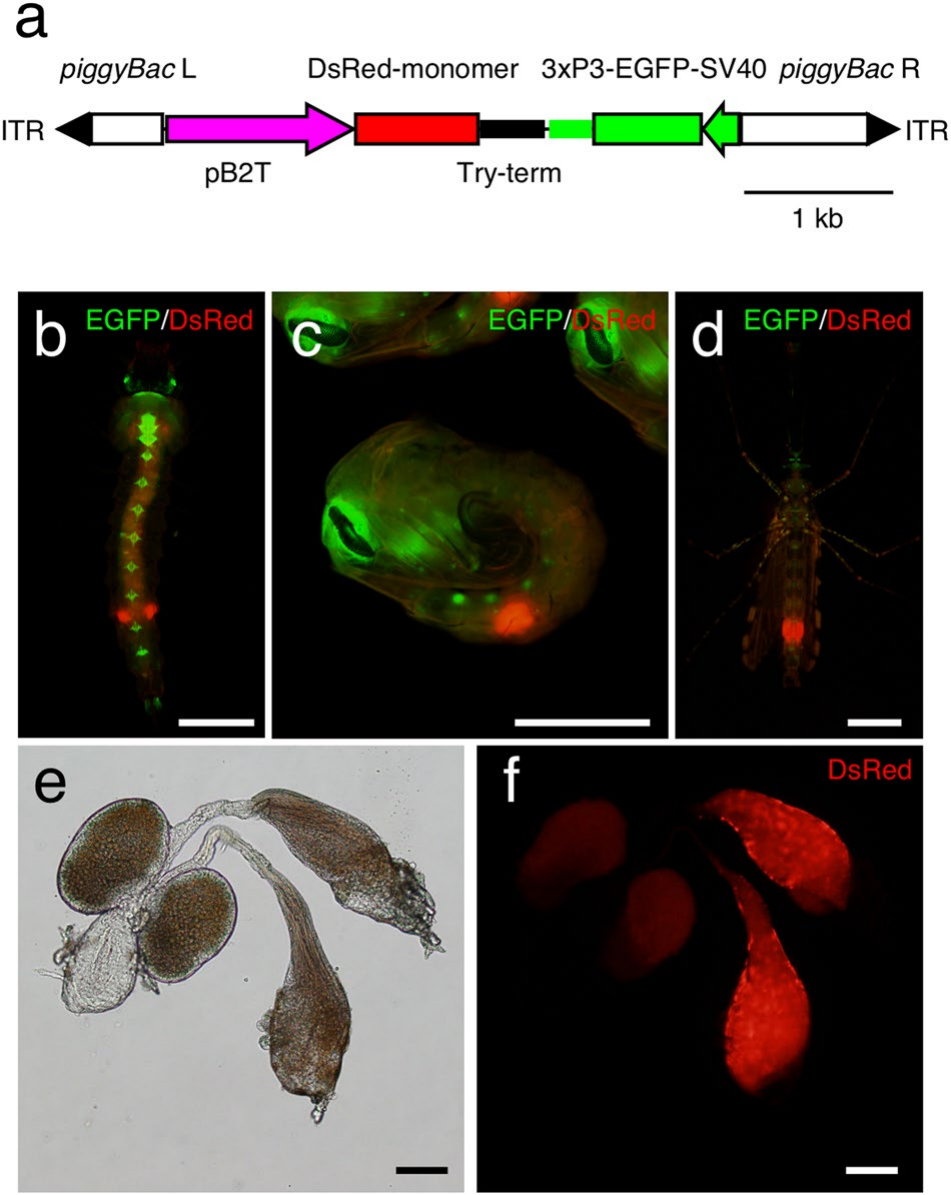
The characterization of B2T-DsRed mosquitoes. (**a**) Gene construct derived from the *piggyBac*-based vector, pBac[pB2T-DsRed; 3xP3-EGFP], which contains a *piggyBac* Left-arm (L) and Right-arm (R) with an inverted terminal repeat (ITR). The *DsRed-monomer* gene is expressed under the control of *An*. *stephensi beta2-tubulin* gene promoter (pB2T) and *An*. *gambiae trypsin* terminator (Try-term). The transformation marker, *EGFP* is expressed under the control of the 3xP3 promoter and SV40 terminator. (**b**) Last instar larva expressing red fluorescence signals in the abdomen of B2T-DsRed mosquito. These larvae were developed to male pupae. (**c**,**d**) Images of pupa and adult B2T-DsRed male mosquitoes, respectively, where the red fluorescence signal was detected only in the sixth abdominal segment. (**e**,**f**) Gonad of B2T-DsRed male mosquito. Red fluorescence signal was detected only in the testis. The panels show the merged fluorescence images. Scale bars = 1 mm (**b–d**), 100 μm (**e**,**f**).

In order to confirm the region of the *Asb2t* promoter responsible for inducing testis specific-expression, we cloned a 2,939 bp fragment including the open reading frame of *Asb2t*, and assembled a *piggyBac*-based transformation vector containing; a gene cassette the putative 5′ regulatory region of *Asb2t* (1,253 bp upstream of *Asb2t* ORF) was placed upstream of a *DsRed-monomer* gene and an *An*. *gambiae trypsin* terminator (Try-term) (Fig. 1a). This transformation vector was injected with *piggyBac* helper plasmid into *An*. *stephensi* embryos, and a transgenic line (B2T-DsRed) was established. In this line, red fluorescence was observed in male pupae and testes of adult males (Fig. 1c–f), whereas no fluorescence was detected in female pupae or adults. Individuals with red fluorescence were observed in the last instar larvae, and then all of them developed to males (Fig. 1b). These results are in agreement with experiments using *b2t* promoters of *An*. *gambiae* and *Ae*. *aegypti*^17,18^. Therefore, the 1,253 bp upstream of the *Asb2t* ORF functions as the promoter of this testis specific-expression of gene of interest.

### Generation of transgenic mosquitoes expressing *mBax* in the testis

**Figure 2:**
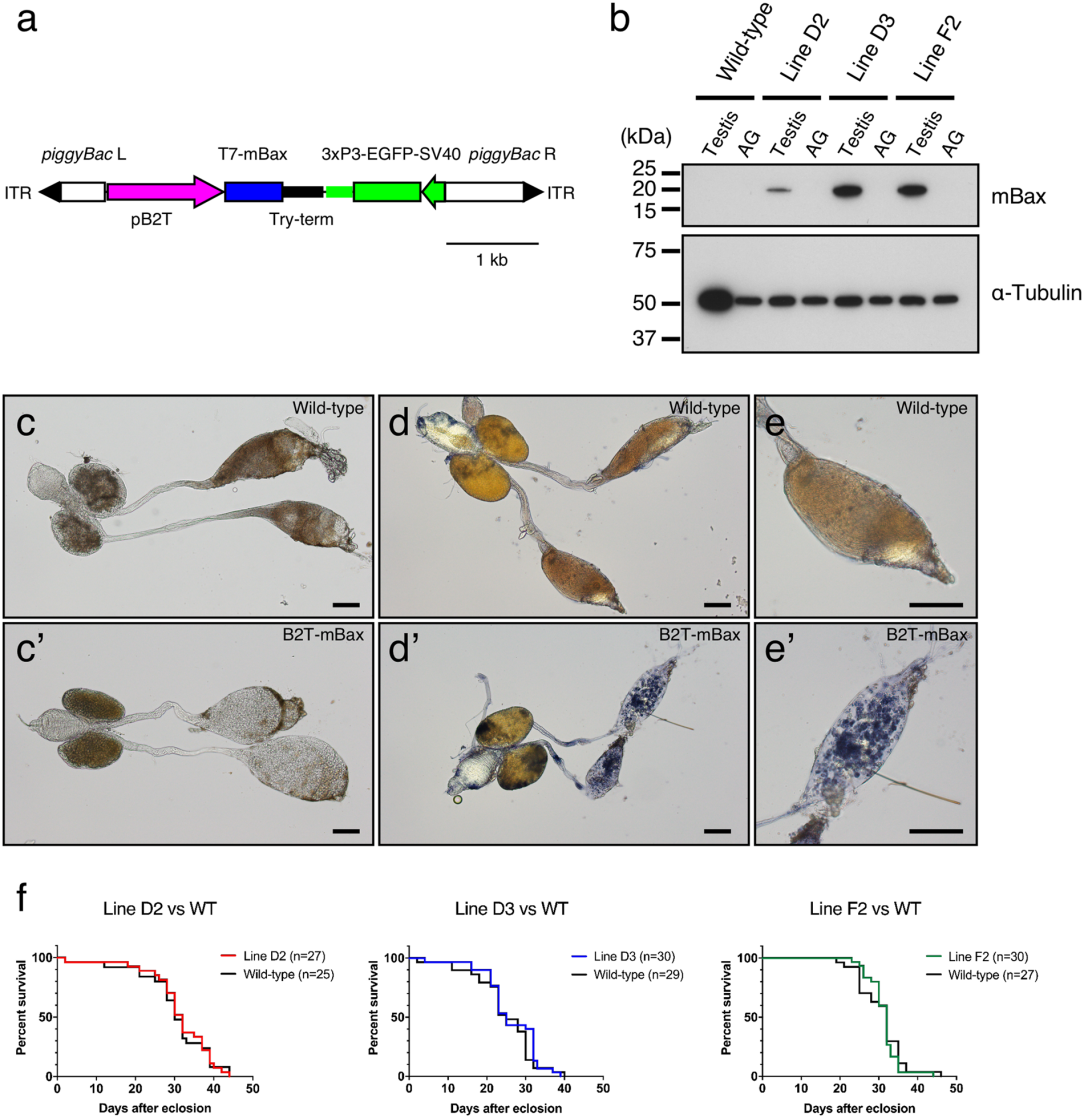
The characterization of B2T-mBax mosquitoes. (**a**) The gene construct derived from the *piggyBac*-based vector, pBac[pB2T-mBax; 3xP3-EGFP]. The *T7-mBax* gene is expressed under the control of *An*. *stephensi b2t* gene promoter and *An*. *gambiae trypsin* terminator. (**b**) Detection of the T7-mBax protein in the testes of B2T-mBax mosquitoes by immunoblotting with anti-T7 antibodies. An anti-alpha tubulin antibody was used as the loading control. Testes and accessory glands of mosquitoes 12-hour post eclosion were used for analysis. (**c**) Gonads of 1-day old adult male wild-type mosquitoes and (**c**’) B2T-mBax mosquitoes. (**d**) Gonads of 1-day old adult male wild-type mosquitoes and (**d**’) B2T-mBax mosquitoes stained with trypan blue. (**e**,**e’**) The magnified images of the indicated testes stained with trypan blue. Scale bars = 100 μm. (**f**) Comparison of survival rates between B2T-mBax and wild-type mosquito males. Mosquitoes immediately after eclosion were used in analyses. Transgenic and wild type mosquitoes were taken from a single heterogeneous strain using EGFP selection, respectively. The survival curves of the groups were estimated by Kaplan-Meier methods. No significant differences were observed between B2T-mBax males and wild-type males. (line D2; *P* = 0.9725, line D3; *P* = 0.4049, and line F2; *P* = 0.6079, calculated by the Log-rank test).

In order to express an *mBax* gene in the testis of *An*. *stephensi*, a *piggyBac*-based transformation vector containing an *mBax* gene with T7-tag under the control of the *Asb2t* promoter and Try-term was generated (Fig. 2a). Three transgenic lines (B2T-mBax, lines D2, D3 and F2) were established (Supplementary Fig. S2). We examined T7-mBax levels in the testes of the B2T-mBax lines using immunoblot analysis with anti-T7 antibody. The T7-mBax protein (Mr = 20 kDa, approximately) was detected in the testes in 12-hour-old males, but not in the accessory glands of each line (Fig. 2b). We then examined the testes in 1-day-old B2T-mBax males. The sperm bundle was not observed in the testes of B2T-mBax mosquitoes, whereas they were observed in wild-type mosquitoes (Fig. 2c and c’). This result suggests that mBax expression causes cell death in spermatocytes, and, as a result, spermatogenesis does not occur normally. In support of this, the testes of B2T-mBax mosquitoes stained with trypan blue, which only stains dead cells (Fig. 2d–e’). This aberrant morphology of the testis was observed in all three transgenic lines, regardless of T7-mBax levels. Together, these data indicate that males of the B2T-mBax mosquito line have no mature sperm and the potential to be sterile.

In order to examine the viability of B2T-mBax males, the survival rates of B2T-mBax adult males and wild-type adult males were monitored. No significant differences were observed between B2T-mBax and wild-type mosquitoes (Fig. 2f, Supplementary Fig. S3). These results indicated that the transgene did not affect the viability of males. Next, we examined the fecundity of B2T-mBax females. Female mosquitoes ingest the blood meal to obtain nutrients for egg reproduction. Following ingestion of the blood meal, yolk synthesis begins and egg maturation is completed. Therefore, heterogeneous females of the B2T-mBax lines that mated with wild-type males were allowed to feed on mice. These females oviposited their eggs, which were then hatched (mean of the hatchability; 76 ± 26–92 ± 6%) (Table 1, Supplementary Fig. S4). Furthermore, in hatched larvae from these females, no significant differences were observed between the number of transgenic individuals (EGFP-positive) and that of wild-type individuals (EGFP-negative). These results indicated that B2T-mBax females were able to produce offspring carrying the transgene. Therefore, B2T-mBax mosquito lines can be maintained by mating between heterozygous transgenic females and wild-type males. Three B2T-mBax lines have been stably maintained for more than 20 generations.

**Table 1:** Evaluation of female fecundity of B2T-mBax mosquitoes.

Genotype (Female)	mBax-line D2	mBax-line D3	mBax-line F2
Number of females that examined mating	30	30	30
Number of females that examined egg laying	30	29	29
Number of females laid eggs (%)	30 (100)	27 (93)	28 (97)
Number of females laid eggs that hatched (%)	30 (100)	26 (90)	27 (93)
Hatchability*, %	92 ± 6	82 ± 17	76 ± 25
Range of hatchability, %	74–99	28–97	3–98
Number of eggs per female*	117.1 ± 28.5	119.6 ± 28.8	99.3 ± 40.5
Range of number of eggs per female	45–168	54–161	7–169
Total number of EGFP positive larvae	1569	1348	1046
Total number of EGFP negative larvae	1674	1288	1088
Two-tailed paired t-test (EGFP positive larvae vs. EGFP negative larvae)	*P* = 0.2000	*P* = 0.2749	*P* = 0.2946

Females (n = 10) were crossed to wild-type males (n = 30).

Three independent experiment results were pooled (Total tested females: n = 30).

*The mean ± SD is shown.

### Males of the B2T-mBax mosquito line are sterile

**Figure 3:**
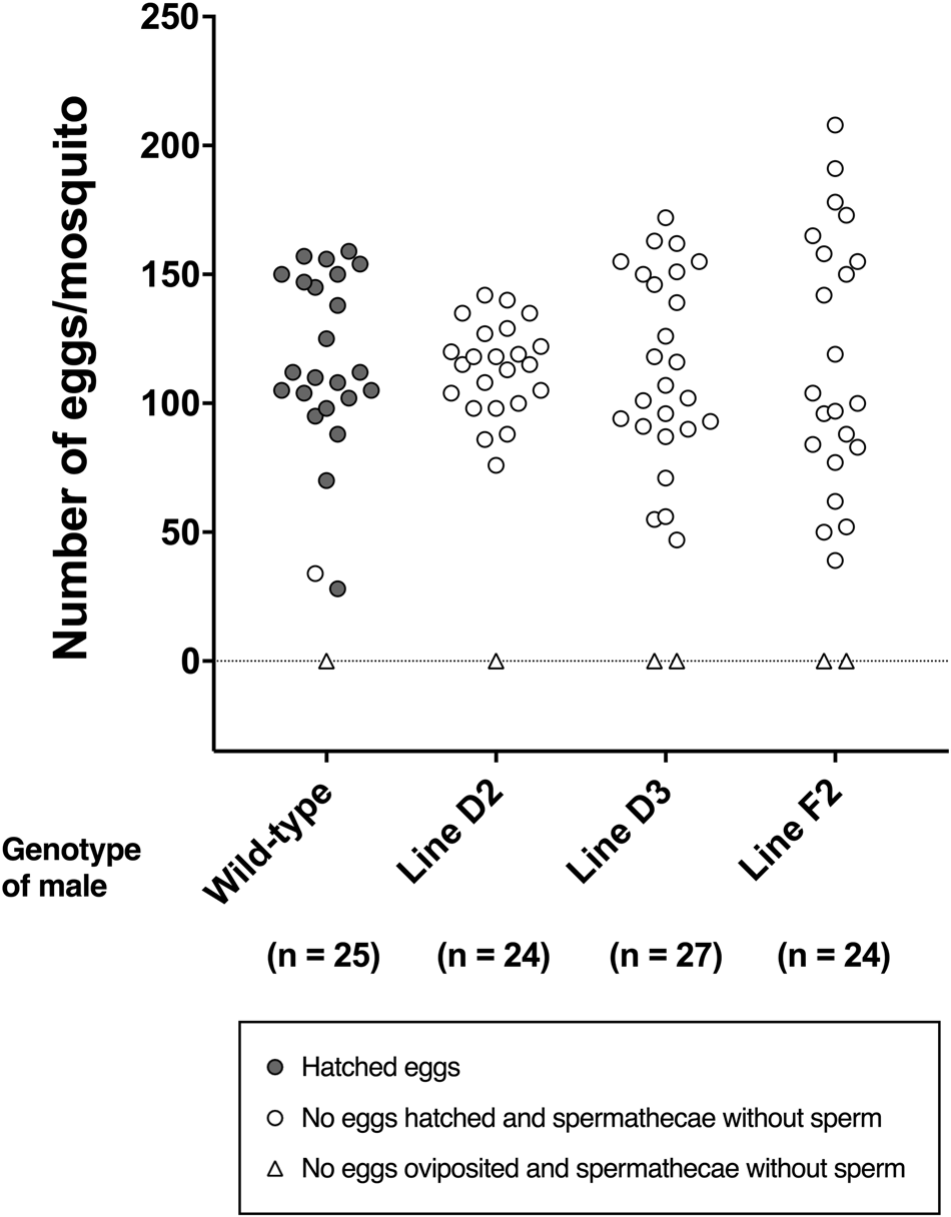
Male sterility of B2T-mBax mosquitoes. Copulated wild-type females with wild type or transgenic males were isolated and collected. Females were allowed to feed on blood within 1–2 day after mating, and then used for the oviposition assay. Each dot corresponds to one female mosquito. Implication of markers is described in the frame.

In order to evaluate of sterility of B2T-mBax males, we isolated the wild-type females that were copulated with transgenic line males or wild-type males, allowed them to feed on mouse blood, and then examined oviposition and egg hatching behaviour. The majority of the females that copulated with transgenic or wild type oviposited eggs. However, only eggs laid by females copulated with B2T-mBax males failed to completely hatch, and these females had no sperm in the spermatheca (Fig. 3, Table 2).

**Table 2:** Evaluation of male sterility of B2T-mBax mosquitoes.

Genotype (Male)	Wild-type	mBax-line D2	mBax-line D3	mBax-line F2
Number of females that examined mating	25	24	27	24
Number of females that examined egg laying	25	24	27	24
Number of females laid eggs (%)	24 (96)	23 (96)	25 (93)	22 (92)
Number of females laid eggs that hatched (%)	23 (92)	0 (0)	0 (0)	0 (0)
Hatchability*, %	91 ± 16	—	—	—
Range of hatchability, %	20–100	—	—	—
Number of eggs per female*	114.7 ± 36.2	113.5 ± 17.5	113.7 ± 36.8	116.9 ± 49.6
Range of number of eggs per female	28–159	76–142	47–172	39–208

This table shows the data summarized in the experiment of Fig. 3.

^*^The mean ± SD is shown.

Moreover, we examined female oviposition and egg hatching behaviour by the mass-mating between thirty wild-type females and thirty transgenic males. Although females placed with B2T-mBax males did not have sperm in the spermatheca, they oviposited eggs, but these eggs did not hatch (Supplementary Fig. S5, Table S1). These results indicate that B2T-mBax line males are completely sterile. On the other hand, eggs laid by females placed with B2T-DsRed line males hatched (Mean of hatchability; 82 ± 23%) (Supplementary Fig. S5, Table S1), and no significant difference was observed in hatchability between females mated with wild-type males and with B2T-DsRed line males. These results suggest that transgene expression in the testis of mosquitoes did not induce the sterility of males, and that the expression of T7-mBax in the testis induces the sterility in males.

### Males of the B2T-mBax mosquito line confer mating refractoriness on females

**Figure 4:**
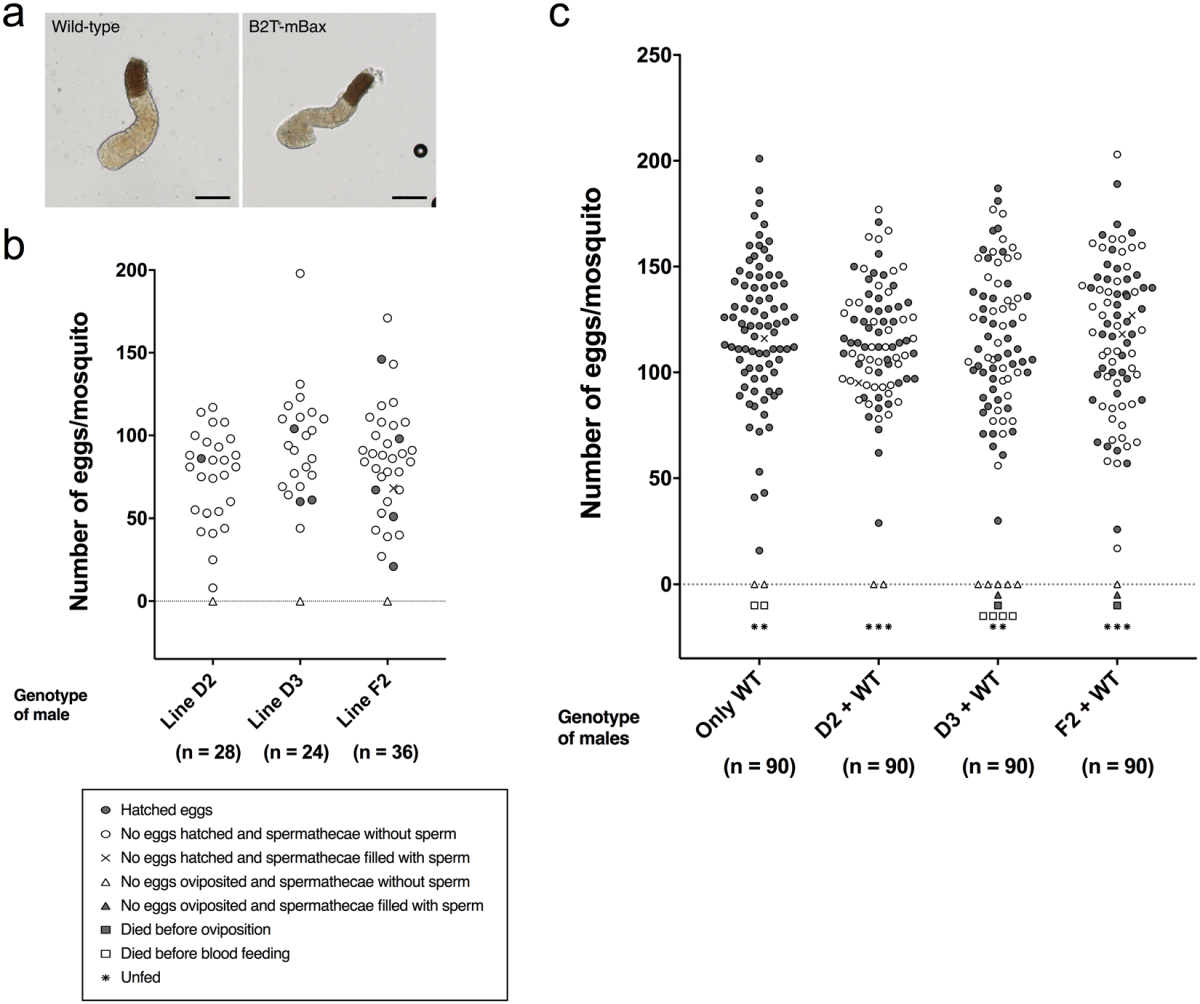
mBax expression in the testis did not affect the mating behaviour of males. (**a**) The mating plug dissected from females mated to B2T-mBax and wild type mosquitoes. Scale bars = 100 μm. (**b**) The mating refractoriness in females by B2T-mBax mosquito males. Copulated wild-type females with B2T-mBax males were isolated and collected. The 8–10 females 2–3 days after mating were pooled, and then placed in cages containing three times wild-type males to the number of females for 1 week. After 1 week, females were allowed to feed blood, and then used for the oviposition assay. Each dot corresponds to one female mosquito. Implication of markers is described in the frame. (**c**) Analysis of the mating competitiveness of B2T-mBax mosquitoes was performed by crossing wild-type females (n = 30) with wild-type males (n = 15) and B2T-mBax males (n = 15) at the same time for 1 week, and then examining the females using an oviposition assay. In these assays, the wild-type males and transgenic males came from two separately raised populations. Each dot corresponds to one female mosquito. Group of only wild type (Only WT) showed that females were placed with wild-type males (n = 15) for 1 week as the control. Implication of markers is described in the frame. Three pooled independent experiments are shown in each line (total tested females; n = 90).

Next, we investigated the response of females copulated with B2T-mBax line males. It is known that mated females are suppressed from further mating by a substance transferred in the mating plug produced by the male accessory glands (MAGs)^33^. Immediately after mating with B2T-mBax line males, the females retained a mating plug and the form appeared normal (Fig. 4a), suggesting the substance of MAGs functions normally to prevent further mating. In order to evaluate whether females mated with B2T-mBax mosquitoes accepted further mating with other males, we monitored wild-type females that had copulated with B2T-mBax line males and were subsequently placed in a cage containing wild-type mosquito males. After 1 week, these females were allowed to feed blood, and then we investigated oviposition and egg hatching behaviour. A small number of females (4–14%) contained sperm in the spermatheca and their oviposited eggs hatched (Fig. 4b, Table 3). All hatched larvae were EGFP-negative, suggesting larvae were progeny of wild-type males. However, in the majority of females, no sperm was observed in the spermatheca, and, although they oviposited eggs, these eggs did not hatch.

**Table 3:** Evaluation of mating refractoriness in females by B2T-mBax mosquito males.

Genotype (1st week male)	mBax-line D2	mBax-line D3	mBax-line F2
Number of females that examined mating	28	24	36
Number of females that examined egg laying	28	24	36
Number of females laid eggs (%)	27 (96)	23 (96)	35 (97)
Number of females laid eggs that hatched (%)	1 (4)	3 (13)	5 (14)
Hatchability*, %	86	62 ± 40	71 ± 21
Range of hatchability, %	—	16–86	41–91
Number of eggs per female*	75.4 ± 27.6	95.4 ± 32.1	84.9 ± 32.9
Range of number of eggs per female	8–117	44–198	21–171

This table shows the data summarized in the experiment of Fig. 4b.

^*^The mean ± SD is shown.

Moreover, we examined the mating refractoriness in females by mass-mating. A small number of females (1–4%) contained sperm in the spermatheca, and all of their progeny were EGFP-negative (Supplementary Fig. S6, Table S2). After mating to wild-type males, a significant difference in the number of females that oviposited fertile eggs was observed when comparing females from the cage without males and those from the cage containing the transgenic males. These results suggest that female mosquitoes mated with B2T-mBax mosquito males were suppressed in further mating, and that B2T-mBax male mosquitoes retain the ability to suppress the female ability to mating further.

### Mating competitiveness of B2T-mBax line mosquito males

Finally, we examined whether B2T-mBax mosquito males were capable of competition with wild-type males for mating. Virgin wild-type females were placed in a cage containing an equal number of transgenic and wild type males, which came from separately raised populations. After one week, we investigated the oviposition behaviour of the females and their egg hatching behaviour. About half of the females (47–52%) oviposited fertile eggs (Fig. 4c, Table 4), and these females contained the sperm in spermatheca. All hatched larvae were EGFP-negative, suggesting the larvae were progeny of wild-type males. Significant differences were observed between the number of females that oviposited fertile eggs in the cage containing only wild-type males and the cage containing the transgenic and wild type males. Similar results were also found by using transgenic and wild type males raised in the same population until pupation (Supplementary Fig. S7, Table S3). These results suggest that B2T-mBax males have the ability to compete with wild-type males for mating under these experimental conditions.

**Table 4:** Evaluation of mating competitiveness of B2T-mBax mosquito males.

Genotype (Male)	Wild-type only	mBax-line D2	mBax-line D3	mBax-line F2
Number of females that examined mating	90	90	90	90
Number of females that examined egg laying	86	87	83	86
Number of females laid eggs (%)	84 (98)	85 (98)	77 (93)	84 (98)
Number of females laid eggs that hatched (%)	83 (97)	45** (52)	43** (52)	40** (47)
Hatchability*, %	83 ± 20	84 ± 22	85 ± 16	85 ± 19
Range of hatchability, %	6–99	1–100	28–99	4–100
Number of eggs per female*	119.8 ± 32.7	115.0 ± 26.0	116.3 ± 33.2	116.7 ± 36.1
Range of number of eggs per female	16–201	29–177	30–187	17–203

This table shows the data summarized in the experiment of Fig. 4c.

*The mean ± SD is shown.

**A significant difference was observed between the number of females that oviposited fertile eggs in the cage containing only wild-type males and in the cage containing the transgenic and wild type males (P < 0.0001, calculated by Fisher’s exact test).

## Discussion

Depletion of the germ cells in male anopheline mosquitoes is reasonable approach to control the population of these disease-spreading insects. In line with this aspect, we assumed that Bax would be a suitable effector gene. Bax is one of the Bcl-2 family proteins and known to function as pro-apoptotic factor. The Bax-mediated mechanism of action has recently been clarified. In mammals, Bax and Bak (Bcl-2 homologues antagonistic killer) form oligomers resulting in pores on the mitochondrial outer membrane, a process known to be involved in apoptosis and the potentiation of interferon response through mtDNA efflux^25,26,34^. Insects have homologues of mammalian Bax; however, it is unclear whether they have same physiological function. Previous study and our recent works found that mouse Bax acts as a pro-apoptotic factor in insects, and that expression of mBax can induce ablation of cells in specific tissues in fly, silkworm, and mosquito^27–29^. In the present study, we newly identified a testis-specific promoter in *An*. *stephensi*, and established lines expressing mBax under the control of this promoter. These transgenic lines, carrying the mBax construct, specifically expressed the gene in the testes. Males of these lines contain aberrant testes without normal sperm. We found that females copulated with B2T-mBax males oviposited eggs, but all of the eggs did not hatch, suggesting that these eggs were not fertilized. Therefore, B2T-mBax line males are completely sterile. In anopheline mosquitoes, oviposition behaviour of females is stimulated by male accessory gland substances transferred during mating^33^. Normal morphology of the male accessory glands in B2T-mBax mosquitoes was observed using microscopic analysis. Our results indicated that the males of B2T-mBax lines retain the ability to stimulate oviposition behaviour in the accessory glands. The substances of the male accessory glands also stimulated the refractoriness of copulated females from further mating^33^. We demonstrated that males of B2T-mBax mosquitoes inhibited the further mating in females. Furthermore, mating plugs similar to those found in females mated with wild-type males were observed in females mated with B2T-mBax males. Therefore, our results indicated that males of B2T-mBax mosquitoes retain normal function of accessory glands. The expression of *mBax* in testes is effective for the sterilization of males and the induction of female sterility for long-term via the mating refractoriness observed in *An*. *stephensi*. For genetic control of *Anopheles* mosquitoes resulting in population suppression, it is imperative that sterile males induce refractoriness to further mating for the lifespan of the female^35^. Another crucial criterion to ensuring the success of genetically induced population suppression is that sterile males must be competitive with wild males for mating^14,16^. In this study, B2T-mBax males appeared to have competitiveness with wild-type males in mating. Since mosquitoes were forced to mate in small cages in this study, further analysis in mass-mating in large spaces would be required for defining the competitiveness of transgenic line males to mate with females in the wild^36,37^.

A previous study reported that spermless males were generated by RNAi-mediated knockdown of a gene involving germ cell differentiation, *zero population growth* (*zpg*), in *An*. *gambiae*^38^. The *zpg*-silenced males were sterile, and females copulated by these males exhibited refractoriness to further mating. The *zpg*-silenced females were also sterile^38,39^, and therefore, a male-specific gene knockout system would be required for development of SIT that targets *zpg* for the induction of sterility. On the other hand, in the present study, our testis-specific mBax expression system induces male-specific sterility in *An. stephensi*. The females of transgenic lines expressing mBax driven by the *b2t* promoter showed fecundity; therefore, these transgenic lines could be maintained by mating between transgenic females and wild-type males. Further studies are required to evaluate whether the expression of mBax affects female fecundity and viability by analyzing the competitiveness of transgenic and wild type females in near-natural environments.

Findings from our previous studies lead us to take note that the *Bax* gene is a remarkable effector for investigation of the functions of particular tissues^27,28^. Here, the great advantage of this genetic system was shown by the induction of complete sterility even in heterozygotes. For the application of our Bax-mediated cell death system, strict regulation of Bax expression is absolutely required because this gene has high pro-apoptotic activity. In this regard, we demonstrated the male-specific sterilization of *An*. *stephensi* by combination of *mBax* and a newly testis-specific tubulin promoter. We accomplished this without affecting basic biological processes of females, and it was suggested that this cell death system is applicable for effective mosquito control. The use of a dominant sterility gene, such as *mBax*, dramatically limits the massive production of males for SIT because the lines can only be maintained through heterozygous females. In terms of both the necessity and direction of the practical development of sterile male strains, it would be expected that a convenient sorting system for males expressing mBax, such as genetic sexing be developed^40–43^. The tetracycline-mediated expression system (tet-off) is preferable for the generation of homozygous individuals^44^. Furthermore, gene regulatory systems, such as site-specific gene integration and gene-drive, are required for further application and practical usage of Bax. Comparative analysis of mBax-expressing males with RIDL and I-*Ppo*I sterile males modelling different scenarios would also be required.

In conclusion, *mBax* is an available effector gene for genetic sterility without affecting other biological processes. Cell death by mBax was inducible in *D*. *melanogaster* as well as *An*. *stephensi*^29^. Since the *b2t* promoter is effective in many dipteran species, this sterilization system is a useful tool for the control of many mosquito species and other dipteran insect pests^17–20^. Efficacy of an mBax-mediated cell death system has been also demonstrated in a silkworm, which is beyond the insect order^28^. This technology promises to provide a versatile tool in the management of beneficial insects as well as in the control of disease vectors and crop pests.

## Methods

### Ethics statement

All mouse procedures were approved by the Institutional Animal Experiment Committee of Jichi Medical University (Number: 16255 and 17142) and in accordance with the Institutional Regulation for Animal Experiments and Fundamental Guidelines for Proper Conduct of Animal Experiment and Related Activities in Academic Research Institutions under the jurisdiction of the Ministry of Education, Culture, Sports, Science and Technology.

### Animals

The *An*. *stephensi* mosquito SDA500 strain was maintained at 26 °C, 60–80% relative humidity, and under conditions of 13-h light/11-h dark at Jichi Medical University. Larvae were fed with fish food, Hikari (Kyorin, Himeji, Japan). Adults were fed on filter paper soaked with a 5% fructose (Nacalai Tesque, Kyoto, Japan). Female BALB/c mice were obtained from Japan SLC (Hamamatsu, Shizuoka, Japan). Mosquito tissues were observed using an inverted microscope, IX73 or SZX7 stereoscopic microscope equipped with a DP73 digital camera (Olympus, Tokyo, Japan).

### Plasmid construction

The *An*. *stephensi beta2-tubulin* gene (*Asb2t*) was amplified by PCR from *An*. *stephensi* genomic DNA using the primers AsB2TPL: ACTGCCGCAGCAGACGGACACATGCCA and AsB2T3’genomeR: GTACACACTCTCTACAGAGCCGGAGCCTTG. The resulting fragments were cloned into a pGEM-T-Easy vector (Promega, Madison, WI, USA) to generate an AsB2T-full plasmid, which was then sequenced.

The DsRed-monomer gene was amplified by PCR from the pDsRed-monomer-C1 plasmid (Takara Bio USA, Mountain View, CA, USA) using the primers DsRedmonoL-NcoI: CAACCATGGACAACACCGAGGACGTCA and DsRedmonoR-SalI: GCGTCGACCTGGGAGCCGGAGTGGC. The resulting DsRed-monomer containing DNA fragment was digested with *Nco*I and *Sal*I, and then cloned into the *Nco*I/*Sal*I site of pBiEx-3 vector (Merck KGaA, Darmstadt, Germany) to generate pBiEx-DsRedmono. Fragments of the DsRed-monomer with an S-Tag and His-Tag were amplified by PCR from pBiEx-DsRedmono using the primers DsRedmonoL-NcoI and pBiExR-NdeI: GGGCATATGTTAGTGATGGTGATGGTGATG and then digested with *Nco*I and *Nde*I. Fragments of the *Asb2t* promoter (pB2T) were amplified by PCR from the AsB2T-full plasmid using the primers AsB2TPL-EcoRI: TTGAATTCACTGCCGCAGCAGACGGA and AsB2TPR-NcoI: GGGCCATGGTTTGCAAACTGCAAACAG and then digested with *EcoR*I and *Nco*I. PCR reactions were performed using KOD plus neo polymerase (Toyobo, Tokyo, Japan). These fragments were cloned into the *Eco*RI/*Nde*I site of pSL-tryter^27^ to generate pSL-pAsB2T-DsRedmono. The pB2T-DsRed-monomer fragment was excised from pSL-pAsB2T-DsRedmono by digestion with *Asc*I and *Fse*I, and was then cloned into the *Asc*I/*Fse*I site of pBac[3xP3-EGFPaf]^45^ to generate the pBac[pB2T-DsRed; 3xP3-EGFP] transformation vector.

The *mBax* gene with a T7-tag was amplified by PCR from a pEF-BOS-T7 vector^46^ using the primers pB2T-mBax-startL: TGCAGTTTGCAAACCATGGCCAGCATGACTGGTGG and TrypolyA-mBax-stopR: GCCGAGATCGCATGCTCAGCCCATCTTCTTCCAGA, and the resulting gene fragment was cloned between pB2T and Try-term of pBac[pB2T-DsRed; 3xP3-EGFP] using the GENEART seamless cloning and assembly kit (Thermo Fisher Scientific, Waltham, MA, USA) to generate the pBac[pB2T-mBax; 3xP3-EGFP] transformation vector. Procedures for the microinjection of vectors into embryos, screening of transgenic individuals, and generation of homozygous lines have been described previously^47^. We used homozygous transgenic B2T-DsRed mosquitoes in all experiments.

### Maintenance of transgenic lines

To maintain B2T-mBax lines, 100–150 heterozygous transgenic females were mated with 100–150 wild-type males in small cages in every generation. After blood meals, wild-type females were allowed to lay eggs in a plastic cup arranged with conically folded filter paper and filled with water at the bottom. Transgenic females expressing EGFP were screened at the pupal stage under a stereoscopic microscope, and then used for mass-mating to obtain the next generation.

### Southern blotting

The isolation of genomic DNA from *An*. *stephensi* and subsequent Southern blot analysis were performed as described previously^48^. Briefly, genomic DNA was digested with *Msp*I, separated on a 0.8% agarose gel, and then transferred to a Hybond-N+ membrane (GE Healthcare UK Ltd., Buckinghamshire, UK). Probe labelling and signals detection were performed using AlkPhos Direct Labelling Reagents and CDP-Star Detection Reagent (GE Healthcare UK Ltd.) according to the supplier’s protocol.

### SDS-PAGE and immunoblotting

A rabbit anti-T7 tag monoclonal antibody (T7 tag, D9E1X XP rabbit mAb, #13246, Cell Signaling, MA, USA) and rabbit anti-alpha-tubulin monoclonal antibody (11H10 mAb, #2125, Cell Signaling) were used as primary antibodies for immunoblotting. Groups of 10 pairs of testes or accessory glands were homogenized by a plastic homogenizer in 100 µl of sample buffer (Nacalai) containing 5% 2-mercaptoethanol and were then boiled at 95 °C for 3 min. Ten microliters of each sample (equivalent to 1 pair of testes or accessory glands) was separated on a 4–12% NuPAGE gel (Thermo Fisher Scientific) and then transferred to an Immobilon-P Membrane (Merck). Membranes were blocked with T-TBS (20 mM Tris-HCl, 137 mM NaCl, pH 7.4, 0.05% Tween-20) containing 5% skimmed milk (Megmilk Snow Brand, Tokyo, Japan). Membranes were incubated with primary antibodies. The polypeptides recognized by primary antibodies were detected with either horseradish peroxidase (HRP)-conjugated goat anti-rabbit IgG (#7074, Cell Signaling). The detection of HRP-labelled antibodies was performed by exposing membranes on Hyperfilm-ECL using ECL Prime Western Blotting Detection Reagents (all GE Healthcare UK Ltd.) according to the supplier’s protocol.

### Reverse transcription-polymerase chain reaction (RT-PCR)

Total RNA was extracted using TRIzol and purified using the PureLink RNA mini kit (all Life Technologies). Five hundred nanograms of total RNA was reverse-transcribed from each sample using the High-Capacity cDNA Reverse Transcription kit (Life Technologies). cDNA was used for the PCR amplification of the *An*. *stephensi b2t* and *An*. *stephensi ribosomal protein S7* (*rpS7*) genes using Taq DNA polymerase (New England Biolabs). The primers AsB2T3′L: 5′-ACAGGTGAATAAACGATGGCCAGCATGACTGGTGG-3′ and AsB2T3′R: 5′-GCCGAGATCGCATGCTCAGCCCATCTTCTTCCAGA-3′ were used to amplify *b2t*. The primers AnsS7L: 5′-GGCGATCATCATCTACGTGC-3′ and AnsS7R: 5′-CGGTCTCTTCTGCTTGTTGG-3′ were used to amplify *rpS7*.

### Isolation of copulated females

One wild-type virgin female 4–7 days after eclosion was entered into a cage containing over 100 wild type or transgenic males 2–7 days post eclosion and after 1–3 hours in the dark. The mating females were observed, and then isolated by an aspirator. Females mating over 20 s were used in subsequent assays. Females 1–2 days after mating were allowed to feed on the mice and were then used for the oviposition assay. The mating plug was observed with under the inverted IX 73 (Olympus) microscope and dissected within 1 hour after mating.

In evaluation of refractoriness to further mating by B2T-mBax line males, 8–10 females were pooled 1–2 days after mating with B2T-mBax males. Then, the females were placed into a cage containing three times the number of wild-type males to females. After 1 week, females were allowed to feed on mice and then used for the oviposition assay.

### Mass mating

Thirty virgin wild-type females 1–2 days after eclosion and thirty virgin males 1–2 days after eclosion from each line were placed in cages (size; 15 cm × 26 cm × 26 cm) and mated for 1 week. After 1 week, females were allowed to feed on mice and then used for the oviposition assay. Male mosquitoes were removed before blood-feedings.

In evaluation of mating refractoriness, wild-type females mated with B2T-mBax males for 1 week, as described above. After 1 week, the males were replaced with thirty virgin wild-type males and then remated for 1 week.

To evaluate the mating competitiveness of B2T-mBax mosquitoes, wild-type females were placed in cages containing fifteen wild-type males and fifteen transgenic males for 1 week, as described above.

To determine fecundity of B2T-mBax mosquito females, ten virgin females and thirty virgin males were placed in cages for 1 week, as described above.

Three independent experiments were performed and pooled in each assay.

### Oviposition assay

Females were individually placed into single plastic cups arranged with a conical folded filter paper and filled with ~30 ml water at the bottom 4–7 days after blood feeding. After 2 days, the spermatheca of females was dissected, and then the presence of sperm was checked. The eggs and hatched larvae were counted. The unhatched eggs were defined as sterile eggs 4 days post laying. In calculation of hatchability, only females that oviposited hatched eggs and more than one larva were used.

### Statistical analysis

All statistical analyses were performed using GraphPad Prism 7 software (GraphPad software, Inc., La Jolla, CA, USA). Comparison of egg hatchability between mating with wild type and transgenic males was performed using a Mann-Whitney *U* test. The number of EGFP positive- and negative-larvae from B2T-mBax females were compared with a two-tailed paired *t*-test. The number of females that oviposited fertile eggs were compared with a Fisher’s exact test. Adult survival curves were generated by Kaplan-Meier methods and compared by the Log-rank test.

### Accession numbers for genes

The following genes were analysed in this study: *mouse Bax* (*mBax*, GenBank NM_007527.3), *An*. *stephensi ribosomal protein S7* (*rpS7*, GenBank AF539918.2).

## Supplementary information

Supplementary Information
