# Supplementary material for: A synthetic male-specific sterilization system using the mammalian pro-apoptotic factor in a malaria vector mosquito
Source: Sci Rep. 2019 Jun 3;9:8160. doi: 10.1038/s41598-019-44480-0 (PMC6547752; doi:10.1038/s41598-019-44480-0)
Supplement: Supplementary file 1 — Supplementary Information [file 41598_2019_44480_MOESM1_ESM.pdf]

Supplementary Information

Title: A synthetic male-specific sterilization system using the mammalian pro-apoptotic factor in a malaria vector mosquito

Authors: Daisuke S. Yamamoto[1, ¶, *], Megumi Sumitani[2, ¶], Katsumi Kasashima[3], Hideki Sezutsu[2], Hiroyuki Matsuoka[1] and Hirotomo Kato[1]

Authors Affiliation:

[1]*Division of Medical Zoology, Department of Infection and Immunity, Jichi Medical University, Yakushiji, Shimotsuke, Tochigi, Japan*

[2]*Transgenic Silkworm Research Unit, Division of Biotechnology, Institute of Agrobiological Sciences, National Agriculture and Food Research Organization (NARO), Owashi, Tsukuba, Ibaraki Japan*

[3]*Division of Functional Biochemistry, Department of Biochemistry, Jichi Medical University, Yakushiji, Shimotsuke, Tochigi, Japan*

[¶]: These authors contributed equally to this work.

[*]: Corresponding author

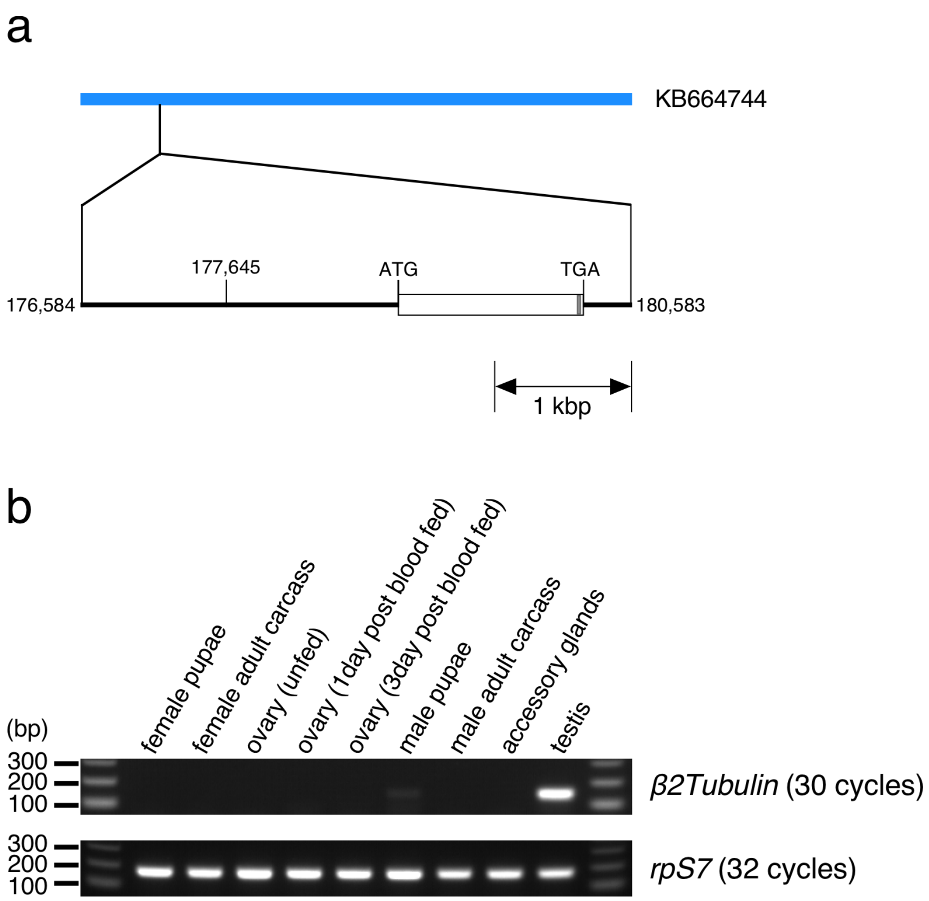

Figure. S1

Characterization of the *beta2-tubulin* gene in *An. stephensi*. (a) Schematic representation of the *beta2-tubulin* gene (*b2t*) structure of *An. stephensi*. The *b2t* gene is located on the scaffold KB664744 of the genomic database of *An. stephensi* in the VectorBase. Box represents exon. Grey band represents the conserved amino acid sequence of testis-specific tubulin in dipteran species. (b) The expression profile of *b2t* mRNA in *An. stephensi*. Pupae and adults (ovary, accessory glands, testis, and carcass) were used in RT-PCR analysis. The *ribosomal protein S7* (*rpS7*) gene was used as a control for ubiquitous expression.

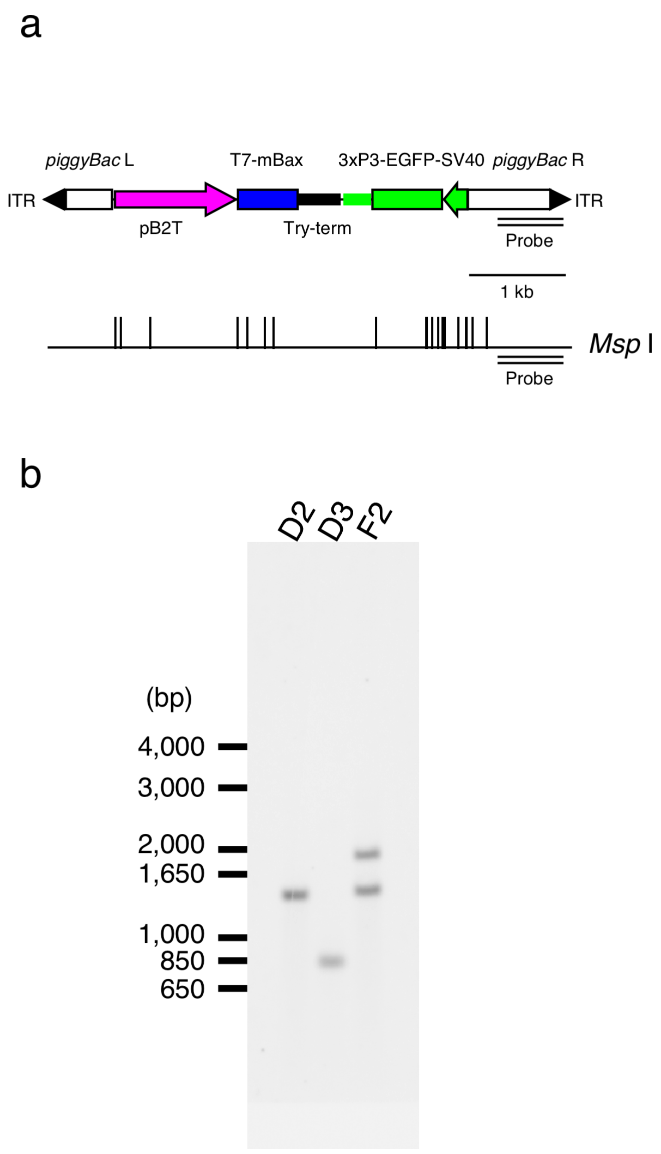

Figure. S2

Southern blot analysis of B2T-mBax lines. The probe region corresponding with *piggyBac* R for analysis is represented by a double line. The restriction enzyme (*Msp*I) site is represented below the scheme of vector. Bottom panel shows Southern blot analysis. Genomic DNA from B2T-mBax mosquito lines (lines D2, D3, and F2) was digested with *Msp*I, hybridized with a probe fragment.

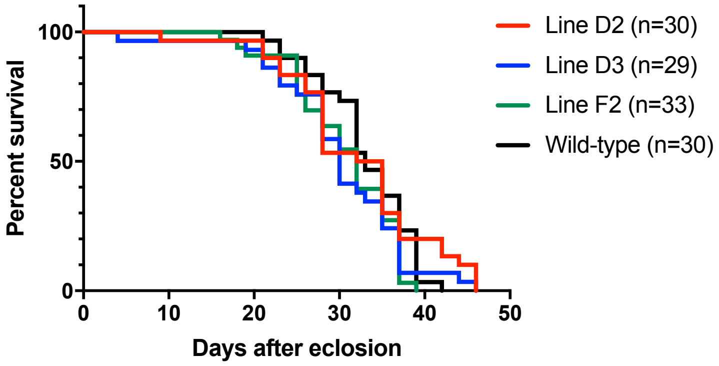

Figure. S3

Comparison of survival rates between B2T-mBax and wild type mosquito males. Transgenic and wild type mosquitoes came from separately raised populations. Therefore, wild-type mosquitoes were not radiated in EGFP excitation light. The survival curves of the groups were estimated by Kaplan-Meier methods. No significant difference was observed between B2T-mBax males and wild-type males. (Line D2 vs. wild type; P=0.6239, Line D3 vs. wild type; P=0.2327, and line F2 vs. wild type; P=0.0633, calculated by the Log-rank test).

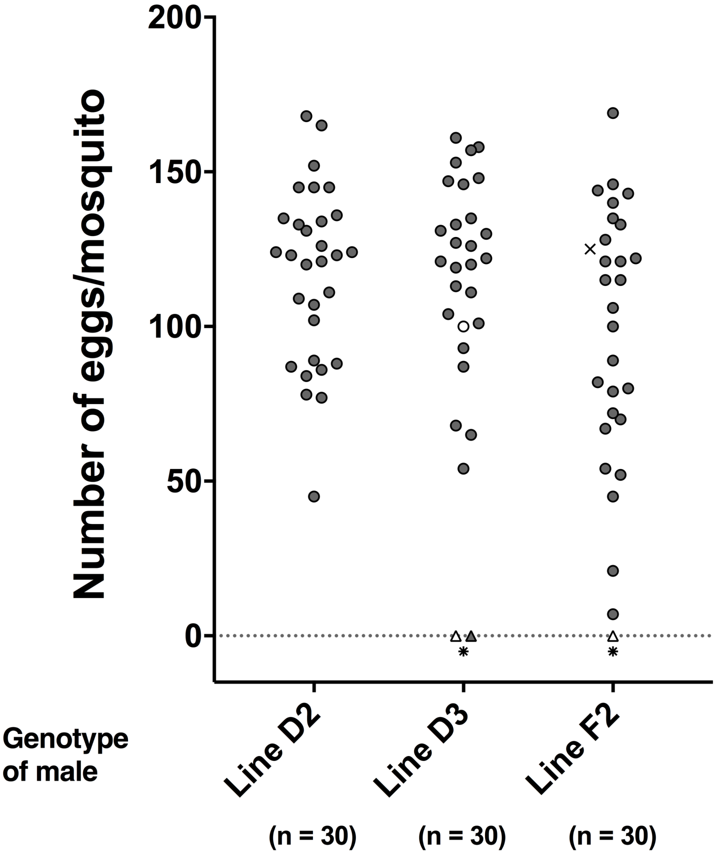

Figure. S4

Analysis of the female fecundity of B2T-mBax mosquitoes by mass mating. B2T-mBax females (n=10) were crossed to wild-type males (n=30) for 1 week. After 1 week, females were fed blood, oviposited, and then dissected for observation of the spermatheca. Oviposited eggs and hatched larvae were subsequently counted. Each dot corresponds to one female mosquito. Implication of markers is described in Fig. S5. Three pooled independent experiments are shown in each line (Total tested females; n=30). Summary of number of females that laid eggs, eggs and hatchability were shown in Table 1.

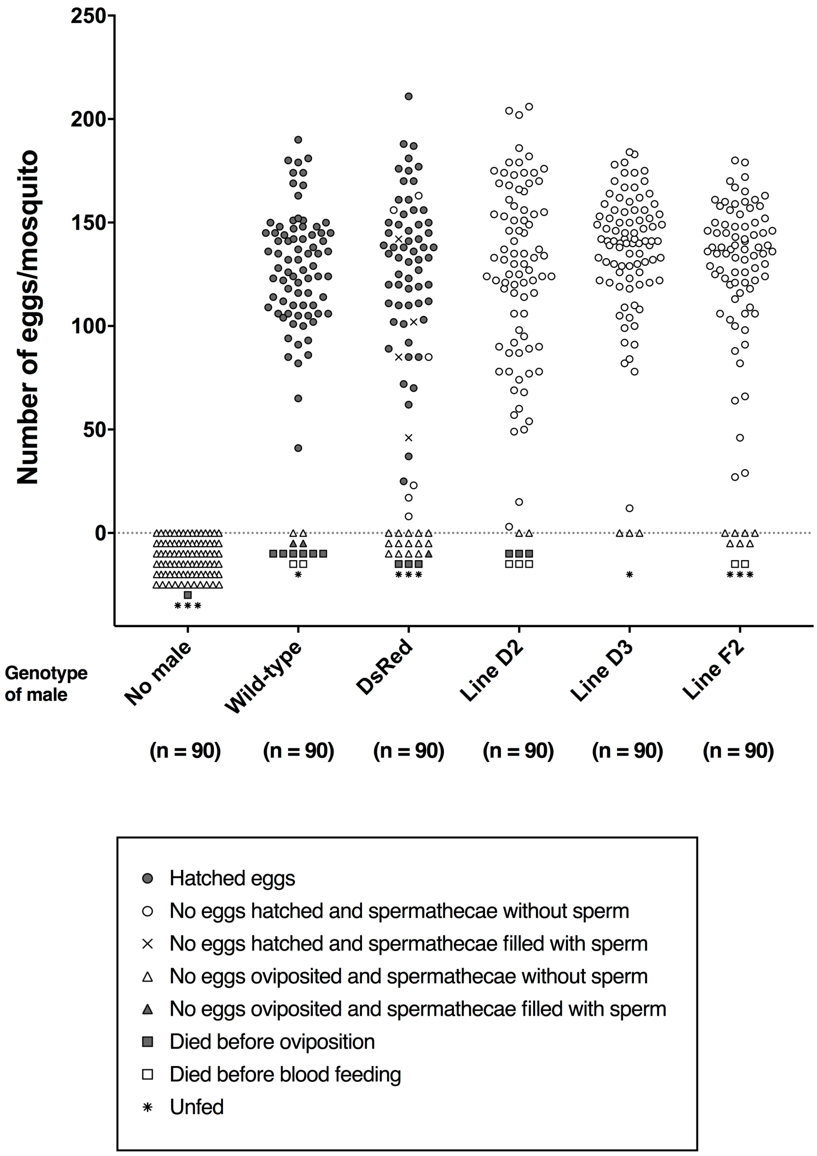

Figure. S5

Analysis of the male sterility of B2T-mBax mosquitoes. Wild-type females (n=30) were crossed to each line of males (n=30) for 1 week. After 1 week, females were fed blood, oviposited, and then dissected for observation of the spermatheca. Oviposited eggs and hatched larvae were subsequently counted. Each dot corresponds to one female mosquito. A group containing no males showed that virgin females, 1-week-old post eclosion, were used for the oviposition assay as a negative control. Implication of markers is described in the frame. Three pooled independent experiments are shown in each line (total tested females; n=90).

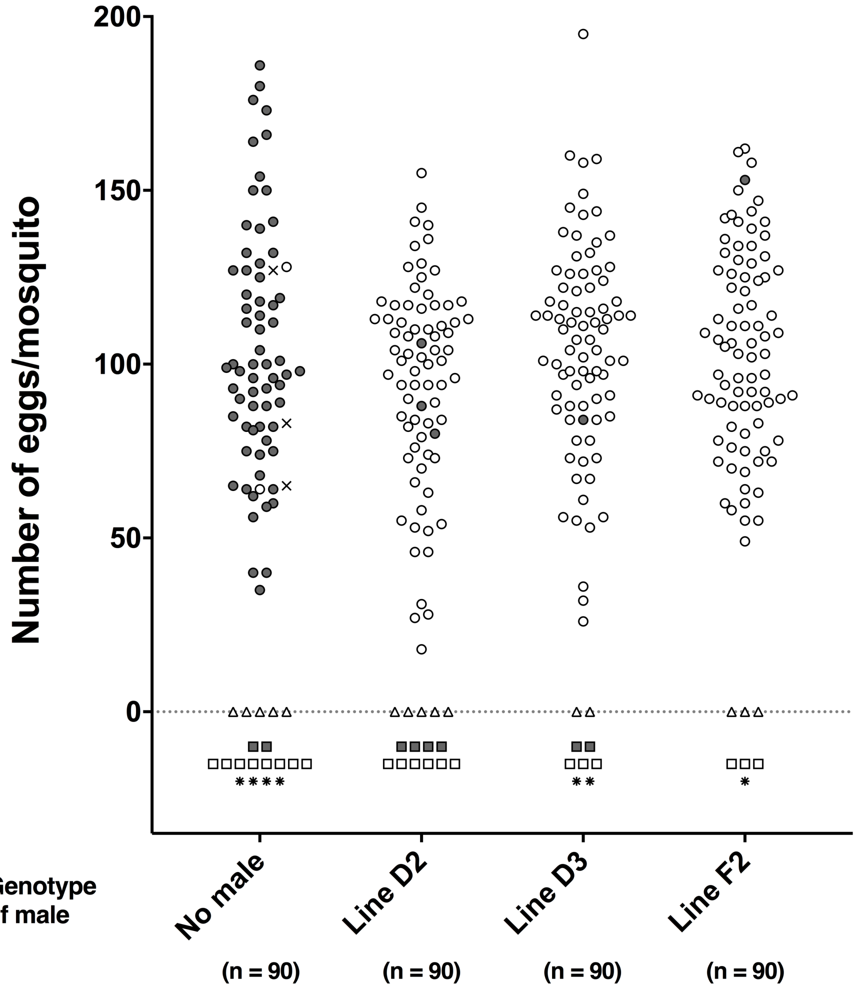

Figure. S6

Analysis of the mating refractoriness in females by B2T-mBax mosquitoes. Wild-type females (n=30) were crossed to each line of males (n=30) for 1 week. After 1 week, the females were placed with wild-type males (n=30) for 1 week, and then examined in the oviposition assay. Each dot corresponds to one female mosquito. A group containing no males and only virgin females 1-week-old post eclosion were mated with wild-type males (n=30) for 1 week and used as the control. Implication of the markers is described in Fig. S5. Three independent pooled experiments are shown in each line (total tested females; n=90).

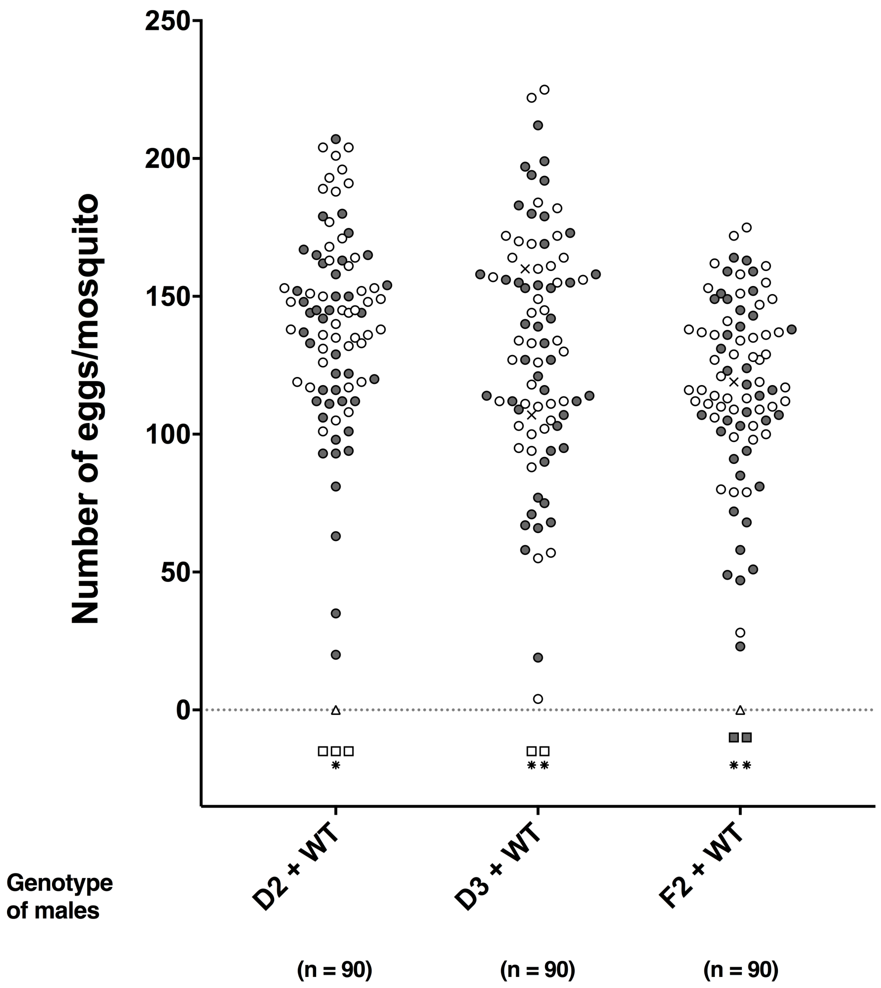

Figure. S7

Analysis of the mating competitiveness of B2T-mBax mosquitoes. Wild-type females (n=30) were crossed to wild-type males (n=15) and B2T-mBax males (n=15) at the same time for 1 week, and then examined in oviposition assay. In this assays, wild-type males and transgenic males were taken from same populations. All larvae were obtained from mating of heterozygous transgenic females and wild-type males. These larvae were grown the same container until last instar, and screened under fluorescent microscopy. Each dot corresponds to one female mosquito. Implication of markers is described in Fig. S5. Three pooled independent experiments are shown in each line (total tested females; n=90).

Table S1. Evaluation of male sterility of B2T-mBax mosquitoes.

Genotype (Male)	None	Wild-type	DsRed	mBax-line D2	mBax-line D3	mBax-line F2
Number of females that examined mating	90	90	90	90	90	90
Number of females that examined egg laying	86	81	84	84	89	85
Number of females laid eggs (%)	0 (0)	77 (95)	69 (85)	82 (98)	86 (97)	78 (92)
Number of females laid eggs that hatched (%)	0 (0)	77 (95)	59 (70)	0 (0)	0 (0)	0 (0)
Hatchability*, %	–	77 ± 27	82 ± 23	–	–	–
Range of hatchability, %	–	2–99	1–100	–	–	–
Number of eggs per female*	–	128.5 ± 27.4	123.3 ± 42.9	127.0 ± 42.8	137.9 ± 27.3	130.8 ± 30.7
Range of number of eggs per female	–	41–190	8–211	3–206	12–184	27–180

This table shows the data summarized in the experiment of Fig. S5.

*The mean ± SD is shown.

**There is no significant difference in hatchability between mating by wild-type male and DsRed male (P = 0.7123, Mann-Whitney test).

Table S2. Evaluation of mating refractoriness in females mated to B2T-mBax mosquito males.

Genotype (1st week male)	None	mBax-line D2	mBax-line D3	mBax-line F2
Number of females that examined mating	90	90	90	90
Number of females that examined egg laying	76	80	83	86
Number of females laid eggs (%)	71 (93)	75 (94)	81 (98)	83 (97)
Number of females laid eggs that hatched (%)	66 (87)	3 **(4)	1 **(1)	1** (1)
Hatchability*, %	79 ± 27	54 ± 48	94	68
Range of hatchability, %	1-100	3–98	–	–
Number of eggs per female*	103.4 ± 34.7	95.3 ± 29.3	104.7 ± 30.3	104.8 ± 28.3
Range of number of eggs per female	35–186	18–155	26–195	49–162

This table shows the data summarized in the experiment of Fig. S6.

*The mean ± SD is shown.

** A significant difference was observed between the number of females that oviposited fertile eggs in the cage containing no males and in the cage containing the transgenic males at 1st week before mating to wild-type males ($P<0.0001$, calculated by Fisher's exact test).

Table S3. Evaluation of mating competitiveness of B2T-mBax mosquito males.

Genotype (Male)	mBax-line D2	mBax-line D3	mBax-line F2
Number of females that examined mating	90	90	90
Number of females that examined egg laying	86	86	86
Number of females laid eggs (%)	85 (99)	86 (100)	85 (99)
Number of females laid eggs that hatched (%)	42** (49)	44** (51)	37** (43)
Hatchability*, %	85 ± 25	91 ± 13	85 ± 17
Range of hatchability, %	3–100	21–100	20–100
Number of eggs per female*	139.9 ± 34.7	131.3 ± 43.5	118.1 ± 32.0
Range of number of eggs per female	35–207	4–225	23–175

This table shows the data summarized in the experiment of Fig. S7.

*The mean ± SD is shown.

** A significant difference was observed between the number of females that oviposited fertile eggs in the cage containing only wild-type males (in Table 3) and in the cage containing the transgenic and wild-type males ($P<0.0001$, calculated by Fisher's exact test).
